# Transcriptomic changes reveal gene networks responding to the overexpression of a blueberry *DWARF AND DELAYED FLOWERING 1* gene in transgenic blueberry plants

**DOI:** 10.1186/s12870-017-1053-z

**Published:** 2017-06-19

**Authors:** Guo-qing Song, Xuan Gao

**Affiliations:** 1 Plant Biotechnology Resource and Outreach Center, Department of Horticulture, Michigan State University, East Lansing, MI 48824 USA; 2 Key Laboratory for the Conservation and Utilization of Important Biological Resources, College of Life Sciences, Anhui Normal University, Wuhu, 241000 China

**Keywords:** Abiotic stress, Cold hardness, C-repeat-binding factor, *DDF1*, Dehydration responsive element-binding factor, Freezing tolerance, *Vaccinium corymbosum*, Woody plant

## Abstract

**Background:**

Constitutive expression of the CBF/DREB1 for increasing freezing tolerance in woody plants is often associated with other phenotypic changes including dwarf plant and delayed flowering. These phenotypic changes have been observed when *Arabidopsis DWARF AND DELAYED FLOWERING 1* (*DDF1*) was overexpressed in *A. thaliana* plants. To date, the *DDF1* orthologues have not been studied in woody plants. The aim of this study is to investigate transcriptomic responses to the overexpression of blueberry (*Vaccinium corymbosum*) *DDF1* (herein, *VcDDF1*-OX).

**Results:**

The *VcDDF1*-OX resulted in enhanced freezing tolerance in tetraploid blueberry plants and did not result in significant changes in plant size, chilling requirement, and flowering time. Comparative transcriptome analysis of transgenic ‘Legacy-VcDDF1-OX’ plants containing an overexpressed *VcDDF1* with non-transgenic highbush blueberry ‘Legacy’ plants revealed the *VcDDF1-*OX derived differentially expressed (DE) genes and transcripts in the pathways of cold-response, plant flowering, DELLA proteins, and plant phytohormones. The increase in freezing tolerance was associated to the expression of cold-regulated genes (CORs) and the ethylene pathway genes. The unchanged plant size, dormancy and flowering were due to the minimal effect of the *VcDDF1-*OX on the expression of DELLA proteins, flowering pathway genes, and the other phytohormone genes related to plant growth and development. The DE genes in auxin and cytokinin pathways suggest that the *VcDDF1-*OX has also altered plant tolerance to drought and high salinity.

**Conclusion:**

A *DDF1* orthologue in blueberry functioned differently from the *DDF1* reported in *Arabidopsis*. The overexpression of *VcDDF1* or its orthologues is a new approach to increase freezing tolerance of deciduous woody plant species with no obvious effect on plant size and plant flowering time.

**Electronic supplementary material:**

The online version of this article (doi:10.1186/s12870-017-1053-z) contains supplementary material, which is available to authorized users.

## Background

The APETALA2/ethylene response (AP2/ERF) transcription factors play a significant role in plant responses to several abiotic stresses (e.g.*,* cold, dehydration, and high salinity) [1]. Accordingly, there have been many recent studies on genome-wide analysis of the AP2/ERF in several plant species, including black cottonwood (*Populus trichocarpa*) [2], *Brassica oleracea* [3], carrot (*Daucus carota* L.) [4], Chinese cabbage (*Brassica rapa ssp. pekinensis*) [5], *Eucalyptus grandis* [6], *Lotus corniculatus* [7], *Medicago truncatula* [8], moso bamboo (*Phyllostachys edulis*) [9], Musa species [10], peach (*Prunus persica*) [11], physic nut (*Jatropha curcas* L.) [12], *Salix arbutifolia* [13], sweet orange (*Citrus sinensis*) [14], and tea (*Camellia sinensis*) [15]. The CBF/DREB1 (C-repeat-binding factor/dehydration responsive element-binding factor 1) genes belong to a large family of AP2/ERF transcription factors and have a conserved DNA binding domain that recognizes the dehydration-responsive element/C-repeat (DRE/CRT) *cis*-acting element in the promoters of their target genes [1, 16, 17]. Studies suggest that all plant species undergo cold acclimation through a similar process that belongs, at least partially, to the CBF/DREB1-mediated cold-response pathway [1, 18–21]. This CBF/DREB1 pathway has been documented in *Arabidopsis thaliana* [16, 22–24]. Additionally, DREB2 transcription factors function in both drought- and heat-stress responses [25–27]. As global warming poses abiotic stresses (e.g., temperature changes and drought) to numerous plant species and threatens the world’s sustainable food production for a growing population, numerous studies have been done to evaluate the potential use of AP2/ERF transcription factors to enhance plant tolerance to abiotic stresses [28].

The usefulness of the CBF/DREB1 pathway genes to enhance freezing tolerance has been demonstrated in both herbaceous and woody plant species [16, 29–31]. However, modulating expression of the CBF/DREB1 pathway genes for enhancing tolerance to abiotic stress is often associated with undesirable changes in plant growth and development [32–35]. For example, the constitutive expression of a peach (*Prunus persica*) *CBF1* gene (*PpCBF1*) in apple rootstock resulted in both improved freezing tolerance and altered plant growth and dormancy [36]. In another study, transgenic grape vines over-expressing a grape (*Vitis vinifera*) *CBF* (*VvCBF4*) showed a slight increase in freezing tolerance in non-cold-acclimated vines and dwarf phenotypes [35]. Similar response for *CBF* overexpression has been reported in *A. thaliana* and other species [32–35]. Ectopic expression of a *CBF1* orthologue from European bilberry (*Vaccinium myrtillus*) enhanced freezing tolerance of *A. thaliana* plants and reduced rosette diameter [37]. Collectively, beyond freezing tolerance, the over-expression of *CBFs* is often associated with reduced plant growth (*CBF1, CBF3,* and *CBF4*) and altered developmental processes such as flowering time, leaf senescence, and plant longevity (*CBF2* and *CBF3*) [24, 32, 33, 35, 38–41]. The occurrence of these additional phenotypic changes is due to the complexity of the *CBFs*-mediated low-temperature regulatory networks and these changes are sometimes considered desirable for crop/fruit production [35, 40, 42, 43]. Of the major CBF/DREB1 transcription factors, the DRE1E_ARATH [designated as *DWARF AND DELAYED FLOWERING 1* (*DDF1*)] and DRE1F_ARATH (designated as *DDF2*) have not been studied in crops [44–48]. *Arabidopsis thaliana* plants overexpressing the *DDF1* showed dark-green leaves, dwarfism, and late flowering; concurrently, the plants displayed enhanced tolerance to cold, drought, heat and high salinity [45, 46, 48].

Overexpression of *CBF2* in *A. thaliana* is mainly associated with delayed leaf senescence and extended plant longevity; additionally, overexpression of *Muscadinia rotundifolia CBF2* gene in *Muscadinia rotundifolia* resulted in growth retardation, dwarfism, late flowering, and abiotic stress tolerance [40, 49]. In this study, we showed a blueberry-derived *CBF* (*BB-CBF*), which was initially considered to be an orthologue of *CBF2* that promoted freezing tolerance in *A. thaliana* [50], was more similar to *A. thaliana DDF1*. Overexpression of the *BB-CBF* (herein renamed as *VcDDF1*) enhanced cold tolerance in leaves and dormant buds but not in flower tissues of a southern highbush blueberry cultivar [51]. Regardless of whether *BB-CBF* is a *DDF1* or *CBF2* orthologue, further studies are needed to facilitate a better understanding of the CBF/DREB1-mediate gene networks in blueberry. Unlike *A. thaliana*, few studies have been conducted to investigate the overall impact of the overexpression of a CBF/DREB1 pathway gene on transcriptomic changes in woody plant species.

Comparative transcriptome analysis is a powerful tool used to identify differential gene expression caused by overexpression of a transgene [52]. For example, overexpression of blueberry *FLOWERING LOCUS T* (*VcFT*) in blueberry plants resulted in plant dwarfing and early flowering [53]. Transcriptome analysis of these transgenic plants revealed differentially expressed (DE) genes in flowering and phytohormone pathway genes that are involved in the phenotypic changes driven by *VcFT*-overexpression [54, 55]. The aim of this study is to elucidate transcriptomic responses to the overexpression of *VcDDF1* (herein, *VcDDF1*-OX) and predict overall performance of *VcDDF1*-OX transgenic blueberry plants. The analysis of DE genes focused on the pathways related to plant growth, flowering or freezing tolerance in blueberry such as plant flowering, CBF-mediated cold/freezing tolerance, phytohormones and DELLA proteins [51, 54, 55].

## Results

### *VcDDF1* and *VcDDF1*-OX in blueberry

**Fig. 1:**
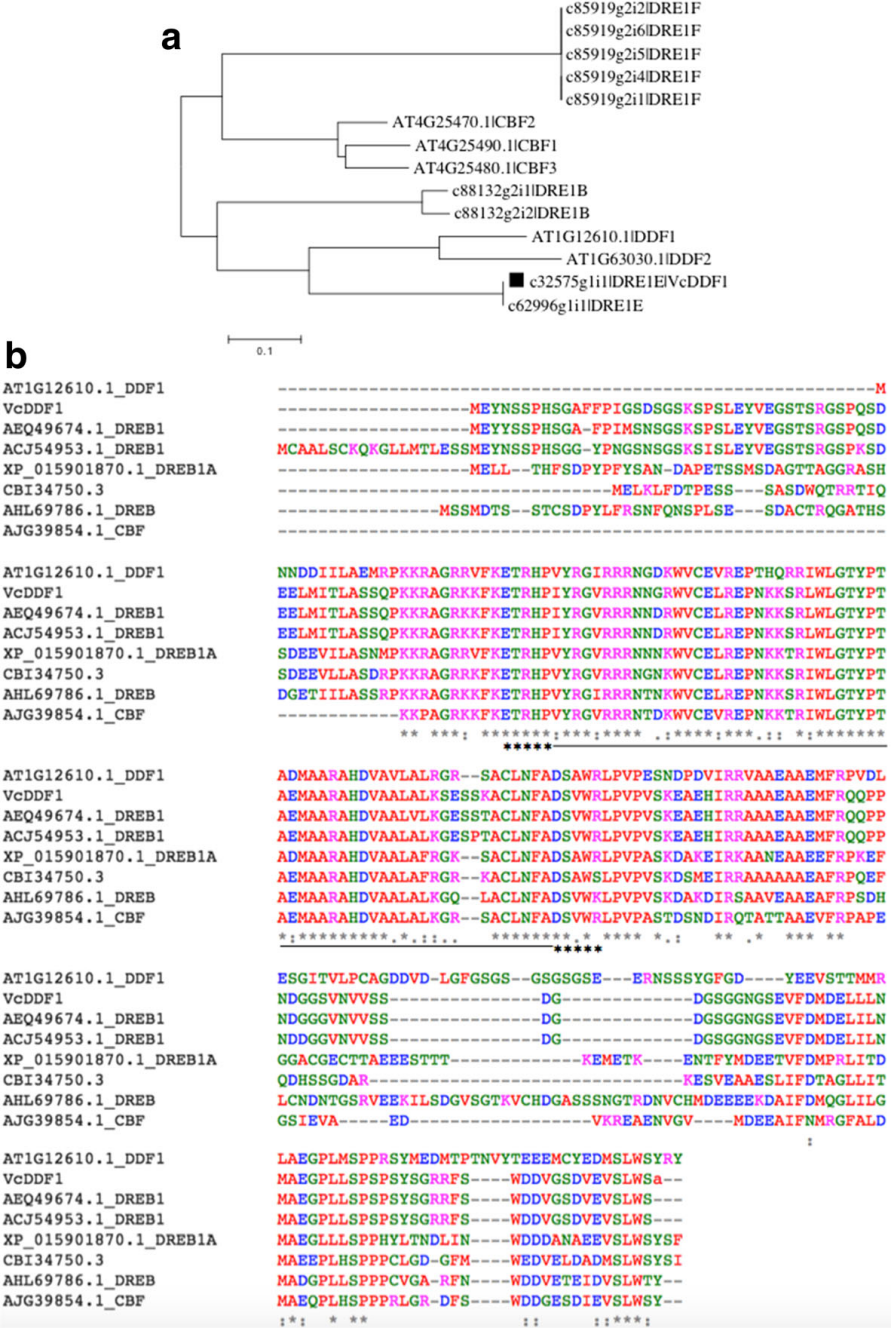
Phylogenetic analysis of CBF/DREB1 proteins of blueberry and multiple protein sequence alignment of DDF1 and VcDDF1 orthologues. **a** Phylogenetic analysis of nucleotide sequences of CBF/DREB1 proteins of blueberry and *A. thaliana* using Neighbor Joining in MEGA 6.06. The bootstrap values were obtained from 500 replicates. The tree was drawn to scale, with branch length equal to substitutions per nucleotide. The black square shows the transgene *VcDDF1* and c62996-g1-i1 is another endogenous *VcDDF1.* **b** Multiple sequence alignment of DDF1, VcDDF1 and protein sequences of VcDDF1 orthologues from six plant species using Clustal Omega (https://www.ebi.ac.uk/Tools/msa/clustalo/). The ethylene-responsive element binding factor/APETELA2 (ERF/AP2) DNA-binding domain is underlined and the [ETRH and DS(A/V)WR] signatures are indicated by *. AEQ49674.1: DREB1 (*Vaccinium myrtillus*). ACJ54953.1: DREB1 (*Vaccinium vitis-idaea*). AHL69786.1: DREB (*Camellia sinensis*). AJG39854.1: CBF (*Actinidia chinensis*). XP_015901870.1: DREB1A (*Ziziphus jujube*). CBI34750.3: unnamed protein product (*Vitis vinifera*)

The *VcDDF1* was initially designated as *BB-CBF* (GenBank: FJ222601.1) due to its similarity to *A. thaliana CBF2*, and this reasoning is valid when *DDF1* is not included in phylogenetic analysis [50]. However, in our recent transcriptome analysis of highbush blueberry using Trinity and Trinotate [56], the *BB-CBF* was annotated as DRE1E_ARATH (*DDF1*). Our designation of *BB-CBF* as *VcDDF1* is the result of the phylogenetic analysis of *A. thaliana* CBF/DREB1 (i.e.*,* CBF1, CBF2, CBF3, DDF1, and DDF2) and the blueberry-derived DRE1E_ARATH, DRE1A_ARATH, DRE1B_ARATH, and DRE1F_ORYSJ, which showed that *BB-CBF* is 52.5% similar to *DDF1* compared 45.9% to *CBF2* (Fig. 1a)*.* The *CBF2* orthologues in blueberry were then assigned to the other two gene contigs (c88132_g2 and c85919_g2 in Fig. 1a). It is interesting to note that *VcDDF1* orthologues in many other woody plants are often annotated as DREB1 due to the conserved ERF/AP2 DNA-binding domains (Fig. 1b).

To investigate the effect of *VcDDF1*-OX at transcript levels in non-acclimated floral buds, comparative transcriptome analysis was conducted in non-transgenic ‘Legacy’ plants and plants of a representative transgenic event ‘Legacy-VcDDF1-OX’ with a single copy of transgenes (named as II7 in our previous report [51]). The ‘Legacy-VcDDF1-OX’ showed a 145-fold increase in the expression of the *VcDDF1* in comparison to the non-transgenic ‘Legacy’ plants. The high *VcDDF1* expression supported our previous observation that the ‘Legacy-VcDDF1-OX’ transgenic event showed high freezing tolerance in electrolyte leakage assays [51].

### Effect of the *VcDDF1-*OX on plant freezing tolerance

**Fig. 2:**
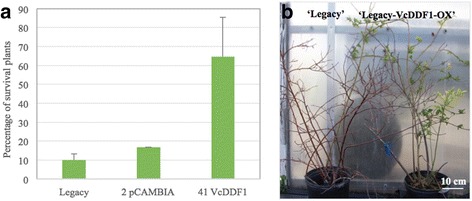
Freezing tolerance in transgenic blueberry plants overexpressing a *VcDDF1*. **a** The survival rates of 3-year old blueberry plants of non-transgenic ‘Legacy’ (*n* = 10), transgenic ‘Legacy-pCAMBIA’ containing a control plasmid pCAMBIA2301 (*n* = 6), and 41 transgenic ‘Legacy-VcDDF1’ events (4–5 plants per event) after the exposure to unprotected environmental conditions in the winters of 2013 and 2014. The representative transgenic event ‘Legacy-VcDDF1-OX’ is included in the 41 events. **b** Under unprotected environmental conditions, one 4-year old ‘Legacy’ plant died and one ‘Legacy-VcDDF1-OX’ plant survived

Constitutive expression of *VcDDF1* resulted in increased freezing tolerance in detached tissues of *A. thaliana* and blueberry plants [50, 51]. In this study, the *VcDDF1-*OX enhanced freezing tolerance in intact plants. The freezing tolerance in (45) four-year plants, one of non-transgenic ‘Legacy’, two of ‘Legacy- pCAMBIA’ events, and 41 of ‘Legacy-VcDDF1’ transgenic events, was investigated. The ‘Legacy-VcDDF1’ transgenic plants showed a significantly higher survival rate (*p* = 0.000126) than those of the non-transgenic ‘Legacy’ and transgenic ‘Legacy-pCAMBIA’ controls (Fig. 2a).

In the winter of 2015, we also investigated the freezing tolerance of 12 three-year plants for both the non-transgenic ‘Legacy’ and the ‘Legacy-VcDDF1-OX’ transgenic event. The ‘Legacy-VcDDF1-OX’ transgenic plants exhibited a higher plant survival rate (83.3%) than the non-transgenic plants (41.7%) (Fig. 2b). Applying a freezing shock of −12 °C for 15 min resulted in visual differences between transgenic ‘Legacy-VcDDF1-OX’ and non-transgenic ‘Legacy’ plants during the plant recovery process. For non-transgenic plant, all leaves and over 90% of the buds showed dying symptoms and died in three weeks. In contrast, for transgenic plants, about 70% of the leaves had no survival leaf tissues, in three weeks; additionally, about 25% buds died. Overall, *VcDDF1-*OX enhanced freezing tolerance of the intact ‘Legacy-VcDDF1-OX’ plants (named as II7 in our previous report).

### Effect of the *VcDDF1-*OX on plant growth and flowering

**Fig. 3:**
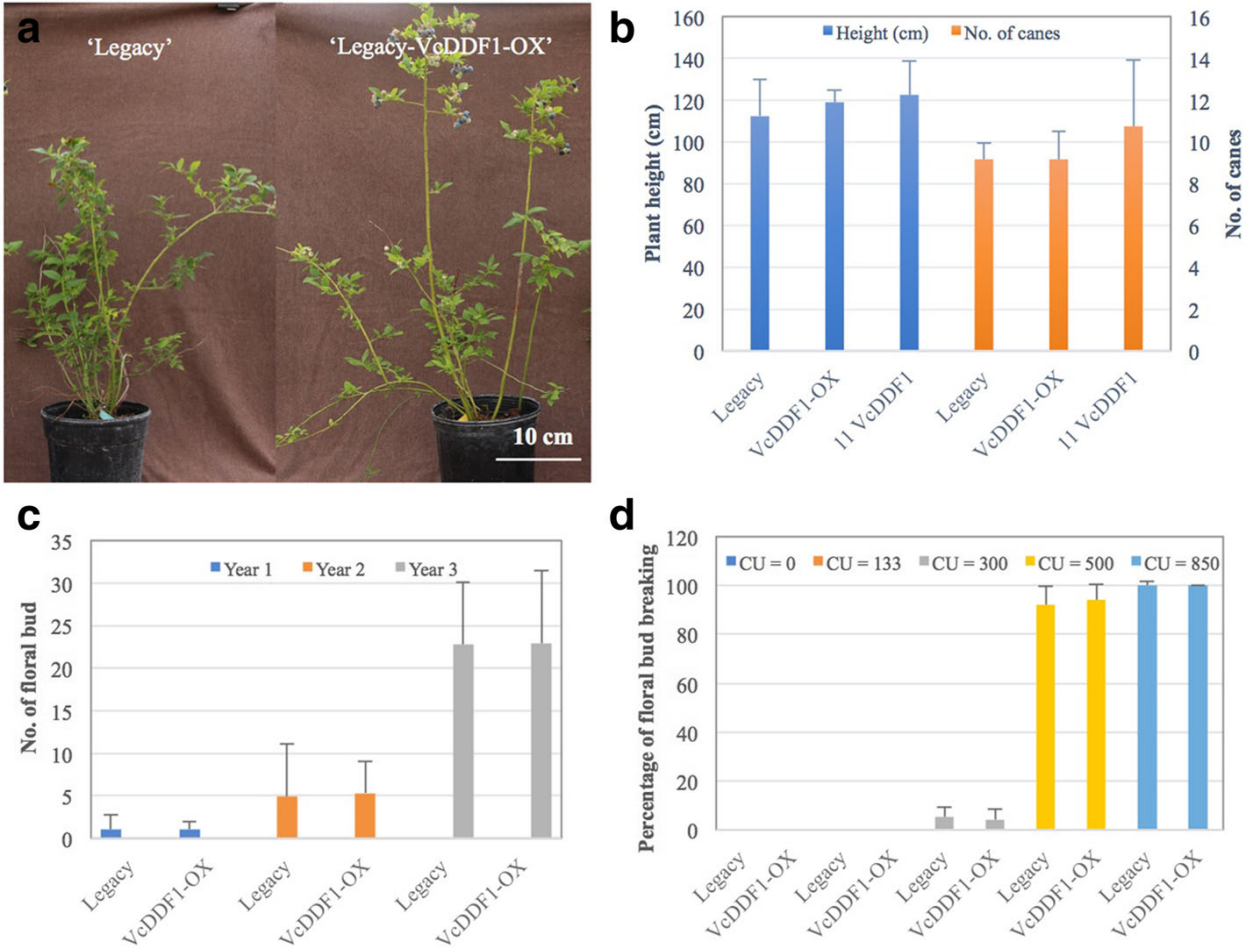
Effect of overexpression of *VcDDF1* on blueberry plant growth and development. **a** Growth of 4-year old plants of ‘Legacy’ and ‘Legacy-VcDDF1-OX’ (herein VcDDF1-OX: a representative transgenic ‘Legacy-VcDDF1’ used for freezing tolerance assay [51] and RNA-seq analysis). **b** Average plant height and number of canes of 4-year old plants. Legacy: non-transgenic southern highbush cultivar ‘Legacy’, three plants. VcDDF1: transgenic ‘Legacy’ containing the *VcDDF1*, three plants for each of the 11 independent transgenic events. VcDDF1-OX: one representative transgenic Legacy-VcDDF1 event, three plants. **c** Average number of floral bud for 1- to 3-year old plants including 12 plants for each of ‘Legacy’ and ‘VcDDF1-OX’. **d** Flowering of four-year old plants (five plants for each treatment) after receiving different amount of chilling. CU: chilling unit

The *VcDDF1-*OX did not alter the growth of transgenic blueberry plants. When four-year transgenic plants of 11 independent ‘Legacy-VcDDF1’ events, including the representative transgenic event ‘Legacy-VcDDF1-OX’, were compared with those of non-transgenic ‘Legacy’ and transgenic control ‘Legacy-pCAMBIA’ plants, all plants looked similar in plant stature and appearance (Fig. 3a) and did not show any difference (*P* < 0.05) in plant height, the number of canes, or the number of flower buds (Fig. 3b, c). These results suggest that *VcDDF1-*OX has little phenotypic effect on blueberry plant growth and floral bud formation. Therefore, *VcDDF1* do not share the designated role of *DDF1* in inducing growth retardation and dwarfism.

In relation to both non-transgenic ‘Legacy’ plants and transgenic control ‘Legacy-pCAMBIA’, delayed flowering was not found in any ‘Legacy-VcDDF1’ plants. For example, ‘Legacy-VcDDF1-OX’ and non-transgenic ‘Legacy’ plants did not show significant differences in the number of floral buds, the age of plant flowering, and the yearly flowering time. Moreover, *VcDDF1-*OX did not affect the chilling requirement of ‘Legacy-VcDDF1-OX’ plants (Fig. 3d). Taken together, *VcDDF1-*OX is not associated with significant changes in plant growth and flowering of tetraploid blueberry plants unlike overexpression of *DDF1* in *A. thaliana* [45, 46].

### Profile of differentially expressed (DE) genes induced by the *VcDDF1-*OX

**Fig. 4:**
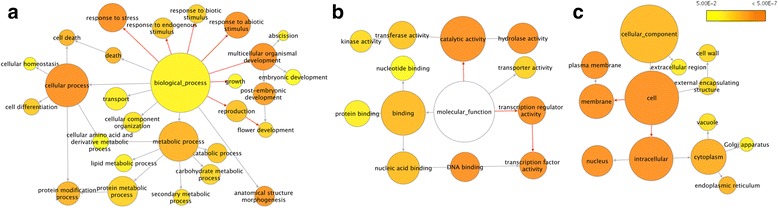
Gene networks of differentially expressed genes in dormant bud tissues of ‘Legacy-DDF1-OX’ plants. The ontology file of GOSlim_Plants in BiNGO was used to identify overrepresented GO terms (*P* < 0.05). Bubble size and color indicates the frequency of GO term and *P*-value, respectively. Red arrows show examples of GO terms related to abiotic resistance, plant growth, and flowering (**a**), the major effect of VcDDF1-OX in molecular function (**b**), and cellular component (**c**)

To reveal the potential roles of the *VcDDF1* at gene transcription levels, comparative transcriptome analysis was conducted between the ‘Legacy-VcDDF1-OX’ and non-transgenic ‘Legacy’ plants. The *VcDDF1-*OX in non-acclimated floral buds of the ‘Legacy-VcDDF1-OX’ plants resulted in 2463 DE genes and 3644 DE transcripts, of which 1668 DE genes were annotated. These DE genes were classified in 54 over-represented Gene Ontology (GO) terms (*P <* 0.05) in the analysis using the GOSlim_Plant as the selected GO file and *A. thaliana* annotation as a reference (Fig. 4). Of the 27 over-represented GO terms in biological_process, two highly over-represented GO terms (i.e.*,* GO:0006950-response to stress and GO:0009628-response to abiotic stimulus) revealed the potential function of the *VcDDF1-*OX in affecting plant freezing tolerance as well as other abiotic stresses. Additionally, two other highly over-represented GO terms (i.e.*,* GO:0007275-multicellular organismal development and GO:0009791-post-embryonic development) suggested that *VcDDF1-*OX could affect plant growth and flower development (Fig. 4a). The *VcDDF1-*OX functions at cellular levels (GO:0005263: cell) and intracellular (GO:0005622: intracellular) levels through its catalytic activity (GO:0003824), DNA binding (GO:0003677), transcription factor activity (GO:0003700), and transcription regulator activity (GO:0030528) (Fig. 4b, c). Overall, the results suggest that *VcDDF1* is a functional DREB1 transcription factor and the *VcDDF1-*OX has an impact on gene expression of multiple pathways in blueberry.

### The responses of blueberry COR genes to the *VcDDF1-*OX

We used 2445 *A. thaliana* COR genes to search for blueberry COR (VcCOR) genes in our blueberry transcriptome reference Reftrinity (GenBank accession number: SRX2728597), which is developed using the RNA sequencing data of leaf, flower, and dormant bud tissues [54]. A total of 24,594 transcripts of 14,231 VcCOR genes showed similarities (e < −20) to 2181 *A. thaliana* COR genes. And 17 transcript contigs of 11 VcCOR genes are the ortholouges of *A. thaliana CBF1/DREB1B*, *CBF2/DREB1C*, *CBF3/DREB1A*, *CBF4/DREB1D*, or *DDF1* (Fig. 1a).

In the dormant buds of ‘Legacy-VcDDF1-OX’ plants, 11,162 DE transcripts of 725 VcCOR genes showed high similarities (e < −20) to 1085 COR genes of *A. thaliana* (Additional file 1: Table S1)*.* Of these DE VcCOR genes, the up-regulated *VcDDF1* and down-regulated *VcCBF2* are the DE CBF/DREB1 genes of blueberry. These results suggest *VcDDF1-*OX regulated some VcCOR genes, which contributed to increase freezing tolerance in intact plants of the ‘Legacy-VcDDF1-OX’ (Fig. 2).

### The responses of blueberry floral genes to the *VcDDF1-*OX

Whereas overexpression of *DDF1* resulted in delaying *A. thaliana* plant flowering [45, 46], *VcDDF1-*OX did not result in visible changes in flowering of tetraploid blueberry plants (Fig. 3c, d). To investigate the potential impact of *VcDDF1-*OX on blueberry flowering, we searched for DE floral genes in the dormant buds of ‘Legacy-VcDDF1-OX’ using the floral gene list of blueberry [54]. Twenty-one floral genes derived from 44 transcripts of 32 gene contigs showed differential expression, of which seven floral genes were up-regulated and 14 were down-regulated (Table 1). This suggests *VcDDF1-*OX affects flowering pathway genes. However, none of the 21 DE floral genes showed changes above four folds. The expression of blueberry *SUPPRESSOR of OVEREXPRESSION OF CONSTANS 1* (*VcSOC1*) was reduced to 67.6% and the expression of blueberry *CONSTANS-LIKE* 5 (*COL5*)-like gene was repressed to 70.5% as much as non-transgenic ‘Legacy’ plants. This down-regulated expression of *VcSOC1* and *VcCOL5* is theoretically associated with delayed flowering [57]. However, the blueberry *SHORT VEGETATIVE PHASE* (*SVP*)(*VcSVP*) showed a decreased expression, which in contrast theoretically promotes plant flowering. In spite of these DE floral genes, the *VcDDF1-*OX was insufficient to promote significant changes in floral bud formation, chilling requirement and flowering time of the ‘Legacy-VcDDF1-OX’ plants (Fig. 3c, d).

**Table 1:** DE floral genes in dormant bud tissues of ‘Legacy-VcDDF1-OX’ plants

Subject id	Floral gene	logFC	logCPM	*P* Value	FDR
c89508_g1_i1	*ABF2*	1.057	2.427	6.59E-07	0.000
c89508_g3_i4	*ABF2*	1.085	1.227	0.000	0.036
c89508_g1_i2	*ABF3*	0.891	2.990	9.35E-06	0.002
c86010_g1_i1	*AGL19*	−0.497	5.315	4.25E-05	0.006
c86010_g1_i2	*AGL19*	−0.605	4.495	0.000	0.017
c94107_g4_i5	*AGL19*	−0.995	1.153	0.001	0.047
c72632_g1_i1	*AGL32*	0.423	7.608	0.000	0.011
c97450_g4_i2	*AP2*	−0.593	3.993	9.23E-06	0.002
c97450_g4_i5	*AP2*	−0.706	2.875	0.001	0.041
c89508_g3_i3	*AREB3*	0.997	1.566	0.000	0.029
c99151_g2_i1	*ARP6*	−0.521	4.522	1.85E-05	0.003
c85121_g1_i1	*ATCOL5*	−0.503	5.503	6.02E-05	0.007
c91872_g2_i3	*CIB1*	−1.396	1.728	8.71E-08	0.000
c92899_g1_i1	*CIB1*	−0.592	3.203	0.000	0.016
c92899_g1_i2	*CIB1*	−0.659	2.907	6.03E-05	0.007
c94404_g2_i1	*CIB1*	−0.525	4.963	6.91E-06	0.001
c94438_g3_i2	*CIB1*	−0.258	8.194	4.55E-05	0.006
c80828_g1_i1	*CKA3*	−0.328	5.530	0.000	0.036
c84766_g4_i1	*CKA3*	−0.389	4.999	0.000	0.024
c88116_g1_i1	*FUL*	−0.402	4.725	0.000	0.036
c91613_g4_i2	*GRF2*	−0.321	6.129	0.001	0.037
c95520_g1_i1	*OsELF3*	−0.639	2.604	0.001	0.054
c95679_g4_i2	*OsELF3*	−0.665	3.706	2.05E-06	0.000
c96650_g1_i1^z^	*OsELF3*	0.610	3.533	0.000	0.027
c96822_g1_i1	*OsELF3*	−0.365	6.469	0.000	0.033
c96828_g2_i2	*OsELF3*	1.577	0.756	1.44E-05	0.002
c85043_g5_i1	*OsGF14e*	1.070	1.501	7.14E-05	0.008
c76027_g1_i1	*PAF1*	−0.496	7.167	7.35E-05	0.009
c91063_g2_i1	*PRR9*	1.141	1.828	6.87E-06	0.001
c86010_g1_i3	*SOC1*	−0.566	4.979	2.65E-06	0.001
c79187_g1_i1	*SPL*	−0.328	6.358	0.000	0.011
c79187_g1_i2	*SPL*	−0.327	6.224	0.000	0.018
c80807_g1_i1	*SPL*	−0.478	6.461	4.00E-08	0.000
c80807_g1_i2	*SPL*	−0.592	5.620	2.05E-10	0.000
c80807_g1_i3	*SPL*	−0.513	6.397	7.63E-11	0.000
c81320_g1_i1 ^y^	*SPL*	0.938	1.576	0.000	0.031
c93310_g3_i1	*SPL*	−0.459	6.681	2.82E-10	0.000
c93310_g3_i2	*SPL*	−0.430	5.768	1.07E-05	0.002
c88116_g2_i1	*SVP*	−0.527	4.975	6.67E-06	0.001
c91377_g1_i14	*SVP*	−1.427	0.741	1.70E-05	0.003
c98453_g2_i3	*TOE1*	−0.680	3.278	3.46E-06	0.001
c98453_g2_i4	*TOE1*	−0.727	2.911	3.54E-05	0.005
c98453_g2_i5	*TOE1*	−0.536	3.998	7.14E-06	0.001
c98453_g2_i7	*TOE1*	−0.455	3.776	0.001	0.052
c87192_g5_i5	*ZmIDS1*	−0.962	1.098	0.001	0.053

^z^also annotated as PCL1_ARATH; ^y^ also annotated as SPL12_ARATH

LogFC: log_2_(fold change) = Log_2_(Legacy-VcDDF1-OX/Legacy). FDR: false discovery rate. LogCPM: Log_2_Count per million reads

### The responses of major phytohormone genes to the *VcDDF1-*OX

**Fig. 5:**
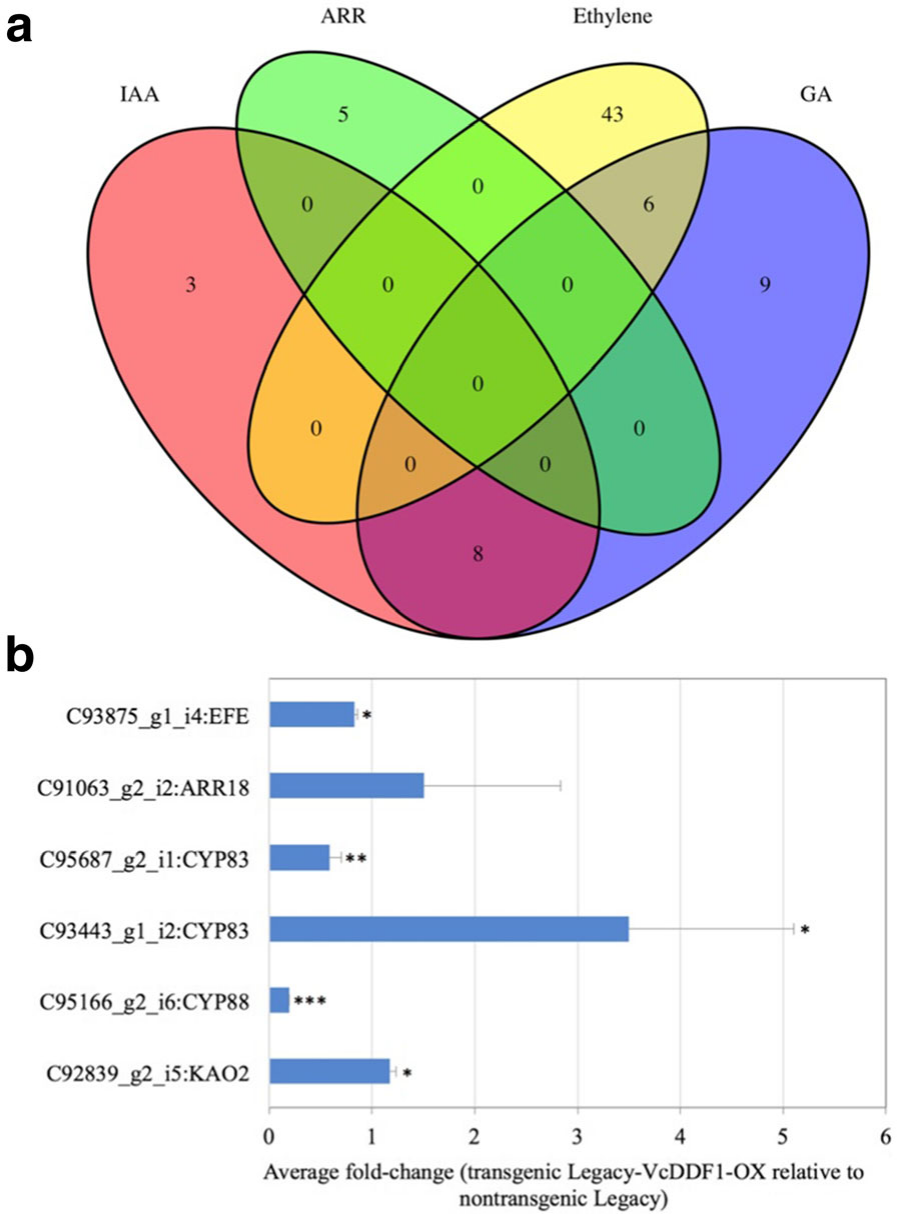
Differentially expressed genes (in comparison to non-transgenic ‘Legacy’) in dormant bud tissues of ‘Legacy-DDF1-OX’ plants. **a** Major phytohormone gene contigs, i.e.*,* gibberellin (GA), abscisic acid (ABA), ARR related genes of cytokinin, and indole acetic acid (IAA). **b** qRT-PCR analysis of representative transcripts. Eukaryotic translation initiation factor 3 subunit H is the internal control. Relative expression (fold-change) in Legacy-VcDDF1-OX was calculated by 2^-∆∆Ct^, ∆∆Ct = (Ct_GOI_ – Ct_nom_)_Legacy-VcDDF1-OX_ – (Ct_GOI_ – Ct_nom_)_Legacy_. Average fold-changes ± standard error of three biological replicates for each of ‘Legacy-VcDDF1-OX’ and ‘Legacy’ plants were plotted. Significant average fold-change determined using a Student’s *t*-test is denoted. An asterisk (*) indicates *p* < 0.05; double asterisks (**) indicate *p* < 0.01; and triple asterisks (***) indicate *p* < 0.001

The *VcDDF1-*OX did not lead to dwarf ‘Legacy-VcDDF1-OX’ plants (Fig. 3a, b), which is inconsistent with the designated function of *DDF1* overexpression in causing dwarf *A. thaliana* plants [45, 46]. To evaluate the potential effect of the *VcDDF1-*OX on plant growth, we identified the DE pathway genes of five major phytohormones [i.e.*,* ABA, GA, auxin (IAA), cytokinin, and ethylene] in dormant buds of ‘Legacy-VcDDF1-OX’ plants. Except for the ABA pathway, DE transcript contigs were found for all other pathways, including 11 for IAA, 23 for GA, five for cytokinin, and 49 for ethylene (Additional file 2: Table S2). The GA pathway has six and eight transcript contigs shared with those in the IAA and ethylene pathways, respectively, indicating the interaction of these pathway genes (Fig. 5a). In the ethylene pathway, 85 out of 86 DE transcripts showed a less than 4-fold change; and only one DE transcript contig of an orthologue of *ETHYLENE-INSENSITIVE5* (*EIN5*) was up-regulated to approximately ten fold. These DE phytohormone genes did not alter plant growth of the ‘Legacy-VcDDF1-OX’ plants (Fig. 3a, b).

### The responses of DELLA proteins to the *VcDDF1-*OX

We found 79 transcript contigs of 47 gene contigs in the blueberry transcriptome reference Reftrinity that show high similarities (e < −20) to the five DELLA protein genes of *A. thaliana*. Of the 79 transcript contigs, two DE transcript contigs of two genes are the *RGL3* orthologues in the bud tissues of the ‘Legacy-VcDDF1-OX’ plants. One of them was repressed to 72.9% and another one was up-regulated to 143.8% (up-regulated by 43.8%). *VcDDF1*-OX poses little effect on the expression of DELLA protein genes in blueberry plants. This provides additional evidence to show the insignificant effect of *VcDDF1-*OX on blueberry plant growth and flowering (Fig. 3).

### Confirmation of the expression of the selected DE transcripts

We designed six pairs of qRT-PCR primers, consisting of two pairs for GA and IAA pathways and one pair for each of cytokinin and ethylene pathways, to validate the DE transcripts of four phytohormone pathways. These selected DE transcripts (FDR < 0.05) often play important roles in their pathways. Of the six DE transcripts tested, qRT-PCR results and RNA-seq data of five transcripts correlated very well (Fig. 5b; Additional file 3: Table S3); only one DE transcript revealed by RNA-seq did not show significant difference (*p* < 0.05) in qRT-PCR analysis (transgenic ‘Legacy-VcDDF1-OX versus non-transgenic ‘Legacy’ samples) but it showed an increase in a regular RT-PCR analysis (Additional file 4: Fig. S1). These results suggest that our RNA-seq data analysis appears reliable for identification of DE genes.

## Discussion

Plant freezing tolerance depends on many factors, such as natural environment, plant species/genotypes, plant developmental stages, acclimation state, organs, and tissues [58]. For woody fruit crops, global warming poses concerns for its impact on the phenology of plant dormancy and freezing tolerance. To address these concerns, a thorough understanding of the genetics and mechanisms of plant freezing tolerance and dormancy is needed. With highbush blueberries, freezing injuries in winter and early spring are major concerns.

### The *CBF/DREB1* orthologues in blueberry

In woody fruit crops, constitutive expression of *CBF1* and *CBF4* or their orthologues has resulted in similar phenotypic changes to those observed in *A. thaliana*, suggesting that *CBF/DREB1* mediated-freezing tolerance is conserved in plants [35, 37, 59, 60]. In this study, our phylogenetic analysis of blueberry CBF/DREB1 proteins suggest the previous *BB-CBF* (an orthologue of *CBF2*) is more likely to be a *DDF1* orthologue (*VcDDF1*) (Fig. 1). The orthologues of this *VcDDF1* are in many deciduous woody plants but none of them was annotated as a *CBF2* orthologue in GenBank (Fig. 1b). It is also interesting that we do not see *CBF1* orthologues in our transcriptome reference. The low coverage of our transcriptome reference may have contributed to the lack of *CBF1* orthologues but the lack of orthologues is probably due to the genome specificity of blueberry plants.

**Fig. 6:**
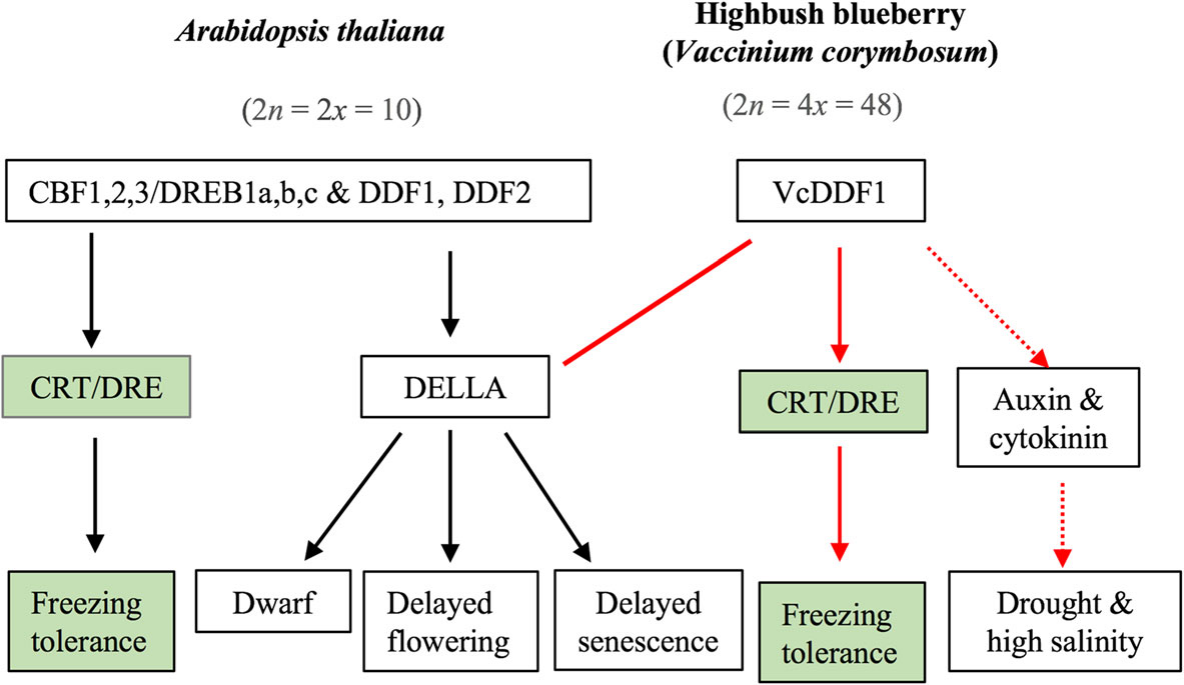
Schematic diagram illustrating the effect of overexpressing a blueberry *DDF1* orthologue on plant freezing tolerance. The diagram illustrating the effect of overexpression of *CBF/DREB1* on freezing tolerance, plant growth and development in *A. thaliana* was derived from previous reports [40, 41, 46]. The overexpression of *VcDDF1* showed little effect on DELLA protein genes but could affect plant tolerance to drought and high salinity through altered gene expression in auxin and cytokinin pathways

Regardless of distinction between *CBF2* orthologue (*BB-CBF*) or *DDF1* orthologue (*VcDDF1*), constitutive expression of this gene is anticipated for dwarfism and late flowering of transgenic plants if its designated function is conserved [45, 46, 49]. However, this is not the phenotypic change observed in transgenic *Arabidopsis* and blueberry plants, where the *VcDDF1-*OX enhanced plant freezing tolerance (Fig. 2; Fig. 3) [50, 51]. These results suggest the function of *DREB1* orthologues in different plant species may vary from their functions designated in *Arabidopsis* (Fig. 6).

### Effect of the *VcDDF1-*OX on plant flowering

In *A. thaliana*, the delayed flowering caused by overexpression of *CBF1,2,3* was due to the increased expression of *FLOWERING LOCUS C* (*FLC*) and repressed expression of *SOC1* [61]. In this study, *VcDDF1-*OX repressed the expression of *VcSOC1*, which is similar to the previous report [61]. However, none of the orthologues of *FLC* in blueberry, including the *MADS-AFFECTING FLOWERING 2*-like gene (*VcMAF2*), *MADS-AFFECTING FLOWERING 5*-like gene (*VcMAF5*), and *VERNALIZATION1-*like gene (*VcVRN1*), showed differential expression. In addition, the expression of *VcSVP*, an orthologue of *A. thaliana SVP* that acts as a negative regulator of *A. thaliana* plant flowering [62], was repressed. These differences between the response of the flowering pathway genes to the *VcDDF1-*OX in blueberry and those of the overexpression of *CBF1,2,3* in *A. thaliana* are responsible for the unchanged flowering phenotype caused by *VcDDF1-*OX.

### Effect of the *VcDDF1-*OX on DELLA protein genes

In *A. thaliana* the delayed flowering and growth retardation caused by the overexpression of *CBF1,2,3* are due to the changes of DELLA proteins [39, 40]. The dwarf *A. thaliana* plants caused by overexpression of *DDF1* was because of reducing bioactive gibberellin [46, 48]. In this study, *VcDDF1-*OX induced little change to the expression of DELLA protein genes, providing further molecular evidence to support that normal growth and flowering of ‘Legacy-VcDDF1-OX’ plants,

### Effect of the *VcDDF1-*OX on phytohormone genes

In *A. thaliana*, the cold-response of *CBF1*, *CBF2*, and *CBF3* is ABA independent while the response of *CBF4* is ABA dependent [41]. Additionally, ethylene signaling can affect expression of *CBFs* [63, 64], and DELLA proteins responding to the overexpression of *CBF/DREB1* genes are GA-related [46, 48]. It seems that *CBF/DREB1* genes interact with phytohormone genes to affect plant growth and development. In this study, *VcDDF1-*OX in tetraploid blueberry plants affected gene expression of the synthesis pathways of IAA, cytokinin, GA, and ethylene but not ABA (Fig. 5a; Additional file 2: Table S2). The 49 DE transcript contigs of 36 gene contigs in the ethylene pathway could contribute to the increased freezing tolerance [63, 64]. The 23 DE transcript contigs of 16 gene in the GA pathway did not show any changes over 4-folds and may have affected the minor changes in the two DE genes of DELLA proteins. Eleven DE transcript contigs of nine genes are orthologues of two IAA pathway genes of *A. thaliana*, including *CYP83B1* and *AUXIN TRANSPORTER PROTEIN* 1(*AUX1*) (c90563_g2_i1); both genes are key regulators of root growth and development [65, 66]. The orthologues of two cytokinin pathway genes *A. thaliana B-TYPE RESPONSE REGULATOR18* (*ARR18*) and *A. thaliana PSEUDO-RESPONSE REGULATOR 2* (*APRR2*) include five DE transcript contigs, the up-regulated *ARR18* orthologue could promote root elongation [67]. The DE transcripts involved in auxin and cytokinin pathways have likely altered plant tolerance to drought or high salinity pending on further investigations [45, 46, 48]. The analysis of DE transcripts of phytohormone genes in addition to DELLA protein genes provide molecular evidence to support that *VcDDF1-*OX was not associated with dwarf and delayed flowering in tetraploid blueberry plants.

### Effect of the *VcDDF1-*OX on freezing tolerance

In terms of its role in freezing tolerance, *VcDDF1* has the same function as *DDF1* and other *CBF/DREB1* genes in *A. thaliana* [48]. Based on both our previous electrolyte leakage assay of in vitro tissues [51] and freezing tolerance assay of intact plants in this study, we have demonstrated that *VcDDF1-*OX is able to enhance freezing tolerance in blueberry plants. In addition, the comparison of VcCOR genes in ‘Legacy-VcDDF1-OX’ plants with non-transgenic ‘Legacy’ has provided molecular evidence to support the role of overexpressed *VcDDF1* in enhanced freezing tolerance (Fig. 2). Of the DE orthologues of *CBF/DREB1* genes, *VcDDF1-*OX down-regulated *VcCBF2*, which did not alter plant growth and flowering.

## Conclusion

In tetraploid blueberry plants, *VcDDF1-*OX resulted in enhanced freezing tolerance and normal plant growth and flowering compared to non-transgenic plants. The increased freezing tolerance is attributed to DE VcCOR genes, which are similar to the *DDF1* and the other *CBF/DREB1* genes (Fig. 6). In contrast to dwarf plant and delayed flowering associated with overexpression of *DDF1* or other *CBF/DREB1* [45, 46, 48], normal phenotypes with regards to plant growth and flowering was due to minimal effect of overexpressed *VcDDF1* on the expression of DELLA proteins, flowering pathway genes, and other phytohormone genes related to plant growth (Fig. 6). The DE genes in phytohormone pathways of auxin and cytokinin imply that *VcDDF1-*OX might enhance plant tolerance to drought and high salinity.

This is the first known investigation of a *DDF1* orthologue in any crop. More importantly, this is the first time the overexpression of a *CBF/DREB1* orthologue was found to enhance plant freezing tolerance without altering plant growth and flowering time. This finding opens a new approach to increase freezing tolerance of deciduous woody plants by using overexpression of *VcDDF1* or its orthologues.

## Method

### Plant materials

A southern highbush blueberry cv. Legacy is tetraploid and needs over 800 chilling units (CU) for normal flowering. The ‘Legacy’ plants used in this study was original derived from the blueberry cultivar collections growing in a research field of the Horticulture Teaching and Research Center of Michigan State University. Transgenic ‘Legacy’ plants (herein ‘Legacy-VcDDF1’) contain a blueberry derived CBF gene (AVI45245.1), which was designated as *BB-CBF* [50, 51] and renamed as *VcDDF1* in this report. The ‘Legacy-pCAMBIA’ is a transgenic control for the *VcDDF1*. ‘Legacy-VcDDF1-OX’ is a representative transgenic ‘Legacy-VcDDF1’ that was used for RNA sequencing. The ‘Legacy-VcDDF1-OX’ (named as II7) contains a single copy of transgenes and showed high freezing tolerance [51]. Production of the ‘Legacy-VcDDF1’ and Legacy-pCAMBIA’ containing the binary vector pCAMBIA2301 was described in our previous report [51].

All non-transgenic and transgenic plants were obtained through micropropagation of in vitro cultured shoots. Plant age was determined based on the time after the shoot was rooted in soil. Rooting of in vitro cultured shoots and plant growth in the greenhouse were performed according the protocols established by Song [68]. All plants were grown normally and were fully vernalized unless otherwise mentioned. For full vernalization in winter, plants were potted and grown in a non-heated hoop house or in a secured courtyard under natural light conditions at Michigan State University, East Lansing, Michigan (latitude 42.701847, longitude −84.482170). The average low and high temperatures in January are −10.6 °C and −1.8 °C, respectively (http://www.usclimatedata.com/climate/east-lansing/michigan/united-states/usmi0248).

### Plant growth and flowering

Four-year old plants were planted in 4-gal pots in 2009 and were grown in a hoop house for winters and were not pruned. These plants included 22 non-transgenic ‘Legacy’, 12 plants of two ‘Legacy-pCAMBIA’ events (6 plants per transgenic event), and 263 plants of 41 ‘Legacy-VcDDF1’ events (5–8 plants/event). Thirty-nine selected plants, including three plants for each of the non-transgenic ‘Legacy’, 11 independent transgenic ‘Legacy-VcDDF1’ events, and one transgenic ‘Legacy-pCAMBIA’ event, were photographed and data was collected twice for plant height, the number of floral buds and the number of canes in October, 2012. The date of early-pink-bud of all plants, defined as the time that the first flower cluster appears, was recorded in the springs of 2009–2012. To test freezing tolerance of intact plants, 4-year old plants including, 10 non-transgenic ‘Legacy’ plants, six plants for each of the two ‘Legacy-pCAMBIA’ events, and 4–5 plants for each of the 41 ‘Legacy-VcDDF1’ events were moved from the greenhouse to a secured courtyard under natural environmental conditions in October of 2013. The number of the survived plants was collected in May of 2015. Freezing tolerance of whole plants were tested by exposing to −12 °C for 15 min in 2012 using actively growing 4-year old plants of one ‘Legacy-VcDDF1-OX’ and one non-transgenic ‘Legacy’. Both plants were then brought to the heated greenhouse with a temperature range of 23 °C - 30 °C under natural photoperiod for recovery. The recovery process was documented through weekly photographs for two months.

Chilling requirement of non-transgenic ‘Legacy’ and transgenic ‘Legacy-VcDDF1-OX’ plants was evaluated with five chilling treatment (i.e.*,* 0, 133, 300, 500, and 850 CU) under controlled conditions in a hoop house in the Horticulture Teaching and Research Center at Michigan State University in the winter of 2012. For each treatment, three ‘Legacy’ and three ‘Legacy-VcDDF1-OX’ plants were used. These plants were three-year old and grown in one-gallon pots. The conversion of selected temperatures to chill units for highbush blueberry was based on the equation: total chill units = 0.5 × number of hours with temperatures below 2.4 °C and 9.2–12.4 °C + 1 × number of hours with temperatures 2.5–9.1 °C – 0.5 × number of hours with temperatures 16–18 °C -1 × number of hours with temperatures above 18 °C [69, 70]. After each chilling treatment, the plants were transferred to a heated greenhouse with a minimum temperature of 23 °C under natural photoperiod. For each plant, the number of floral buds was counted and the dates of early-pink-bud and petal fall stages were recorded. The number of the unopened floral buds was counted after eight weeks.

New non-transgenic ‘Legacy’ and transgenic ‘Legacy-VcDDF1-OX’ plants were developed through micropropagation in January of 2012 for further investigations. Twelve plants for each of the non-transgenic ‘Legacy’ and transgenic ‘Legacy-VcDDF1-OX’ were investigated from 2013 to 2016. These plants were grown in one-gallon pots in the courtyard. The number of floral buds was counted and the dates of the early-pink-bud and petal fall stages were recorded yearly. The number of fruit clusters was counted in July 2016.

### RNA preparation, sequencing, and de novo transcriptome assembly

Floral buds were collected in November 2013 before the plants were exposed to a non-heated greenhouse for chilling treatments. All tissues collected were frozen immediately in liquid nitrogen and stored at −80 **°**C.

Total RNA isolation, RNA sequencing using the Illumina HiSeq2500 platform, de novo transcriptome assembly using the Trinity platform (trinity/20140413p1) [56] were described in our recent report [54].

### Differential expression analysis and transcriptome annotation

RNA-seq reads of three biological replicates for each of ‘Legacy’ and ‘Legacy-VcDDF1-OX’ were analyzed. Two technical replicates were sequenced for each biological replicate and were combined together for analysis. The paired reads, two sets for each biological replicate, were aligned to the transcriptome reference developed for ‘Legacy’ [54] and the abundance of each read was estimated using the Trinity command “align_and_estimate_abundance.pl”. The Trinity command “run_DE_analysis.pl --method edgeR” was used for differential expression analysis. The differentially expressed (DE) (relative to non-transgenic ‘Legacy’ unless other mentioned) genes or transcripts with false discovery rate (FDR) values below 0.05 were used for further analyses. Transcriptome annotation was performed using Trinotate_v2.0 (https://trinotate.github.io).

### Phylogenetic analysis of *VcDDF1*

Representative nucleotide sequences of CBF/DREB1 of five *A. thaliana CBF/DREB1* genes were retrieved using The *A. thaliana* Information Resource (TAIR) server (https://www.*arabidopsis*.org/tools/bulk/index.jsp). Orthologues of *A. thaliana CBF/DREB1* genes in blueberry were identified from our annotated transcripts. The selected transcripts were converted to amino acid sequences based on BLAST results retrieved using the NCBI server (http://blast.ncbi.nlm.nih.gov/Blast. cgi). Selected nucleotide sequences from both *A. thaliana* and blueberry were aligned using Clustal Omega multiple sequence alignment program at EBI with default parameters (http://www.ebi.ac.uk/Tools/msa/clustalo/). Phylogenetic trees were generated using MEGA 6.06 software [71].

The VcDDF1 protein sequence (AVI45245.1) was used to search for VcDDF1 orthologues using the NCBI server. The selected protein sequences were aligned using Clustal Omega multiple sequence alignment program at EBI with default parameters.

### Gene network construction

Annotated transcripts were imported to Cytoscape 3.4.0 under BiNGO’s default parameters with selected ontology file ‘GOSlim_Plants’ and selected organism *A. thaliana* [72, 73].

### Identification of the selected pathway genes

Representative protein sequences of selected genes of *A. thaliana* were download from the TAIR server (https://www.arabidopsis.org/tools/bulk/sequences/index.jsp). The retrieved sequences were used to search the transcriptome reference of blueberry (herein refTrinity) using the tblastn command of BLAST+. The resultant transcripts that show e-value lower than −20 were used to screen the DE transcript list of non-acclimated floral buds.

The 2637 cold-regulated genes (CORs) identified in wild-type *A. thaliana* plants and 172 CORs differentially expressed at a warm temperature (22 °C) in transgenic *A. thaliana* plants overexpressing *CBF1*, *CBF2* or *CBF3* were obtained from Park et al. [42]. These CORs were used to identify their orthologues in blueberry (VcCORs), which was used to analyze the effect of *VcDDF1-*OX on VcCORs. The blueberry floral genes identified in our previous study [54] were used to analyze flowering pathway genes affected by *VcDDF1-*OX*.*

The pathway genes of major phytohormones [i.e.*,* gibberellin (GA) [74], abscisic acid (ABA) [75], cytokinin [76], indole acetic acid (IAA) [77], and ethylene [78]] in *A. thaliana* were retrieved from TAIR_10 server based on published gene identities (Additional file 5: Table S4). In addition, sequences of *A. thaliana* DELLA proteins were used to analyze the effect of *VcDDF1-*OX*.* Five *A. thaliana* DELLA proteins (Additional file 5: Table S4), including GIBBERELLIC ACID INSENSITIVE (*GAI*) (AT1G14920.1), REPRESSOR OF GA1 (*RGA1*) (AT2G01570.1), RGA-like 1 (*RGL1*) (AT1G66350.1), RGA-like 2 (*RGL2*) (AT3G03450.1), and RGA-like 3 (*RGL3*) (AT5G17490.1), were used to search for the DELLA protein genes in blueberry.

#### Quantitative RT-PCR (qRT-PCR) of DE transcripts

Reliability of DE genes/transcripts identified through RNA-seq was evaluated through qRT-PCR analysis of six selected transcripts (Additional file 3: Table S3). These transcripts are from the representative DE genes in auxin, ethylene, cytokinin, and GA pathyways. They have high fold changes (>2) and sequence specificity (based on alignment result of different isoforms) for PCR amplification. Eukaryotic translation initiation factor 3 subunit H was the internal control (Additional file 3: Table S3).

The RNA samples used for RNA-sequencing, including samples of three biological replicates for each of ‘Legacy’ and ‘Legacy-VcDDF1-OX’, were used for cDNA preparation. Reverse transcription of RNA to cDNA was performed using SuperScript II reverse transcriptase (Invitrogen, Carlsbad, CA, USA). The resulting cDNA of one micro gram of RNA was diluted (volume 1: 4) in water and 1 μl/sample (25 ng) was used for PCR reactions.

The primers were designed using the online tool provided by Integrated DNA Technologies, Inc. (https://www.idtdna.com/Primerquest/Home/Index), where the primers were synthesized (Additional file 3: Table S3). qRT-PCR was performed in triplicate on an Agilent Technologies Stratagene Mx3005P (Agilent Technologies, Santa Clara, CA) using the SYBR Green system (Life Technologies, Carlsbad, CA). In each 25 μl reaction mixture, 25 ng cDNA, 200 nM primers and 12.5 μl of 2× SYBR Green master mix were included. The reaction conditions for all primer pairs were 95 °C for 10 min, 40 cycles of 30 s at 95 °C, 60 s at 60 °C and 60 s at 72 °C, followed by one cycle of 60 s at 95 °C, 30 s at 55 °C and 30 s at 95 °C. The specificity of the amplification reaction for each primer pair was determined by the melting curve. Transcript levels within samples were normalized to the eukaryotic translation initiation factor 3 subunit H. Fold changes were calculated using 2^-∆∆Ct^, where ∆∆Ct = (Ct_GOI_ – Ct_nom_)_Legacy-VcDDF1-OX_ – (Ct_GOI_ – Ct_nom_)_Legacy_ for each transgenic ‘Legacy-VcDDF1-OX’ versus a non-transgenic ‘Legacy’ sample (*n* = 3) [79]. In addition, regular RT-PCR was also used for selected transcripts. The reaction conditions using 50 ng cDNA per reaction for all primer pairs were 94 °C for 2 min, 35 cycles of 45 s at 94 °C, 60 s at 60 °C and 60 s at 72 °C, with a final 10 min extension at 72 °C. RT-PCR products were separated on 1.0% agarose gel containing ethidium bromide, visualized, and photographed under UV light.

## Additional files

Additional file 1: **Table S1.** Differentially expressed cold-regulated genes (CORs) of blueberry in non-acclimated floral buds. FDR (false discovery rate) < 0.05. LogFC: log_2_(fold change) = Log_2_(Legacy-VcDDF1-OX/Legacy). *Some of these transcripts show similarities to multiple Arabidopsis-gene-ids that are not listed in this table (XLSX 138 kb)

Additional file 2: **Table S2.** Differentially expressed phytohormone genes (transgenic ‘Legacy-VcDDF1-OX’ vs. non-transgenic ‘Legacy’ plants) in dormant bud tissues of blueberry plants. LogFC: log_2_(fold change) = Log_2_(Legacy-VcDDF1-OX/Legacy) (DOCX 116 kb)

Additional file 3: **Table S3.** Primers used for RT-PCR. FDR (false discovery rate) < 0.05. LogFC: log_2_(fold change) = Log_2_(Legacy-VcDDF1-OX/Legacy) (DOCX 76 kb)

Additional file 4: **Fig. S1.** RT-PCR analysis of differentially expressed transcripts in leaf tissues of non-transgenic ‘Legacy’ and transgenic ‘Legacy-VcDDF1-OX’. *Eukaryotic translation initiation factor 3 subunit H* is the internal control (JPEG 119 kb)

Additional file 5: **Table S4.** The pathway genes of major phytohormones [i.e.*,* gibberellin (GA) [75], abscisic acid (ABA) [75], cytokinin [76], indole acetic acid (IAA) [77], ethylene [78], and DELLA protein genes in *A. thaliana* (DOCX 89 kb)

## Abbreviations

ABA: Abscisic acid
AP2/ERF: APETALA2/ethylene response (AP2/ERF) transcription factors
CBF/DREB1: C-repeat-binding factor/dehydration responsive element-binding factor 1
CORs: Cold-regulated genes
CPM: Count per million reads
DDF1: *DWARF AND DELAYED FLOWERING 1*
DE: Differentially expressed
FDR: False discovery rate
GA: Gibberellin
GO: Gene ontology
IAA: Indole acetic acid
OX: Overexpression
qRT-PCR: Quantitative reverse transcriptase polymerase chain reaction
