# Supplementary material for: Transcriptomic changes reveal gene networks responding to the overexpression of a blueberry DWARF AND DELAYED FLOWERING 1 gene in transgenic blueberry plants
Source: BMC Plant Biol. 2017 Jun 19;17:106. doi: 10.1186/s12870-017-1053-z (PMC5477172; doi:10.1186/s12870-017-1053-z)
Supplement: Supplementary file 2 — Table S2. Differentially expressed phytohormone genes (transgenic ‘Legacy-VcDDF1-OX’ vs. non-transgenic ‘Legacy’ plants) in dormant bud tissues of blueberry plants. LogFC: log2(fold change) = Log2(Legacy-VcDDF1-OX/Legacy) (DOCX 116 kb) [file 12870_2017_1053_MOESM2_ESM.docx]

**Table S2.** Differentially expressed phytohormone genes (transgenic ‘Legacy-VcDDF1-OX’ vs. non-transgenic ‘Legacy’ plants) in dormant bud tissues of blueberry plants. LogFC: log_2_(fold change) =Log_2_(Legacy-VcDDF1-OX/Legacy).

|  | Transcrip_id: annotation | LogFC_Bud | LogCPM_Bud |
| --- | --- | --- | --- |
| Auxin |  |  |  |
|  | c79196_g2_i1:_CYP83B1,_SUR2,_RNT1,_RED1,_ATR4 | 0.763 | 2.027 |
|  | c90563_g2_i1:_AUX1,_WAV5,_PIR1,_MAP1 | 1.151 | 1.176 |
|  | c91808_g6_i1:_CYP83B1,_SUR2,_RNT1,_RED1,_ATR4 | 0.662 | 6.648 |
|  | c93443_g1_i1:_CYP83B1,_SUR2,_RNT1,_RED1,_ATR4 | -1.524 | 1.573 |
|  | c93443_g1_i2:_CYP83B1,_SUR2,_RNT1,_RED1,_ATR4 | -1.799 | 1.322 |
|  | c93443_g1_i3:_CYP83B1,_SUR2,_RNT1,_RED1,_ATR4 | -1.368 | 1.789 |
|  | c95518_g1_i1:_CYP83B1,_SUR2,_RNT1,_RED1,_ATR4 | 0.197 | 7.601 |
|  | c95687_g3_i1:_CYP83B1,_SUR2,_RNT1,_RED1,_ATR4 | 1.168 | 1.503 |
|  | c97488_g1_i2:_CYP83B1,_SUR2,_RNT1,_RED1,_ATR4 | -0.687 | 4.546 |
|  | c98942_g1_i8:_CYP83B1,_SUR2,_RNT1,_RED1,_ATR4 | 0.970 | 1.644 |
|  | c99070_g3_i1:_CYP83B1,_SUR2,_RNT1,_RED1,_ATR4 | 0.541 | 4.671 |
|  |  |  |  |
| GA | Transcrip_id: annotation | LogFC_Bud | LogCPM_Bud |
|  | c54669_g1_i1:_GA5,_GA20OX1,_AT2301,_ATGA20OX1 | -1.022 | 1.628 |
|  | c77450_g1_i1:_YAP169,_GA20OX3,_ATGA20OX3 | 0.413 | 4.908 |
|  | c79196_g2_i1:_GA3,_CYP701A3,_ATKO1 | 0.763 | 2.027 |
|  | c83619_g1_i1:_YAP169,_GA20OX3,_ATGA20OX3 | 0.634 | 6.758 |
|  | c86276_g1_i1:_KAO2 | 0.954 | 7.151 |
|  | c91808_g6_i1:_GA3,_CYP701A3,_ATKO1 | 0.662 | 6.648 |
|  | c92062_g3_i2:_YAP169,_GA20OX3,_ATGA20OX3 | 1.750 | -0.057 |
|  | c92839_g2_i5:_KAO2 | -1.687 | 1.394 |
|  | c93443_g1_i1:_GA3,_CYP701A3,_ATKO1 | -1.524 | 1.573 |
|  | c93443_g1_i2:_GA3,_CYP701A3,_ATKO1 | -1.799 | 1.322 |
|  | c93443_g1_i3:_GA3,_CYP701A3,_ATKO1 | -1.368 | 1.789 |
|  | c93875_g1_i2:_YAP169,_GA20OX3,_ATGA20OX3 | 0.860 | 2.572 |
|  | c93875_g1_i3:_YAP169,_GA20OX3,_ATGA20OX3 | 0.718 | 2.803 |
|  | c93875_g1_i4:_YAP169,_GA20OX3,_ATGA20OX3 | 1.298 | 2.528 |
|  | c95166_g2_i1:_KAO2 | 1.531 | 2.230 |
|  | c95166_g2_i2:_KAO2 | 1.141 | 2.269 |
|  | c95166_g2_i4:_KAO2 | 0.864 | 2.374 |
|  | c95166_g2_i6:_CYP88A3,_ATKAO1,_KAO1 | 1.683 | 2.032 |
|  | c95518_g1_i1:_GA3,_CYP701A3,_ATKO1 | 0.197 | 7.601 |
|  | c96066_g1_i1:_GA4H,_ATGA3OX2,_GA3OX2 | 1.329 | 1.495 |
|  | c97397_g2_i1:_KAO2 | 0.882 | 3.642 |
|  | c97488_g1_i2:_GA3,_CYP701A3,_ATKO1 | -0.687 | 4.546 |
|  | c99070_g3_i1:_GA3,_CYP701A3,_ATKO1 | 0.541 | 4.671 |
|  |  |  |  |
| Cytokinin | Transcrip_id: annotation | LogFC_Bud | LogCPM_Bud |
|  | c91063_g2_i1:_ARR18,_RR18 | -1.141 | 1.797 |
|  | c91063_g2_i2:_ARR18,_RR18 | -1.355 | 1.828 |
|  | c95267_g3_i1:_APRR2 | 0.487 | 4.920 |
|  | c95267_g3_i2:_APRR2 | 0.423 | 4.828 |
|  | c95267_g3_i5:_APRR2 | 0.469 | 4.823 |
|  |  |  |  |
| Ethylene | Transcrip_id: annotation | LogFC_Bud | LogCPM_Bud |
|  | c100210_g1_i3:_CTR1,_SIS1,_AtCTR1 | -0.637 | 3.477 |
|  | c100313_g2_i1:_CTR1,_SIS1,_AtCTR1 | 0.368 | 5.668 |
|  | c100313_g2_i2:_CTR1,_SIS1,_AtCTR1 | 0.407 | 5.722 |
|  | c63618_g1_i1:_AIN1,_EIN5,_XRN4,_ATXRN4 | -3.320 | -0.596 |
|  | c72531_g1_i1:_CTR1,_SIS1,_AtCTR1 | -2.115 | -0.184 |
|  | c74528_g1_i1:_EIL2 | -1.289 | 0.985 |
|  | c75581_g1_i1:_LACS8 | -1.924 | 0.339 |
|  | c77450_g1_i1:_EFE,_ACO4,_EAT1 | 0.413 | 4.908 |
|  | c78420_g1_i2:_EFE,_ACO4,_EAT1 | 1.360 | 0.613 |
|  | c78550_g1_i2:_CTR1,_SIS1,_AtCTR1 | 1.237 | 1.885 |
|  | c78650_g1_i1:_RAN-1,_RAN1,_ATRAN1 | 0.460 | 5.079 |
|  | c82685_g1_i1:_RAN-1,_RAN1,_ATRAN1 | 0.429 | 5.605 |
|  | c82685_g1_i2:_RAN-1,_RAN1,_ATRAN1 | 0.529 | 5.382 |
|  | c82685_g1_i3:_RAN-1,_RAN1,_ATRAN1 | 0.355 | 5.864 |
|  | c83619_g1_i1:_EFE,_ACO4,_EAT1 | 0.634 | 6.758 |
|  | c84182_g1_i3:_AIN1,_EIN5,_XRN4,_ATXRN4 | -1.117 | 1.560 |
|  | c85411_g2_i6:_CTR1,_SIS1,_AtCTR1 | -1.413 | 1.249 |
|  | c85611_g2_i1:_CTR1,_SIS1,_AtCTR1 | 1.166 | 0.931 |
|  | c86338_g2_i1:_CTR1,_SIS1,_AtCTR1 | 0.689 | 2.777 |
|  | c86338_g2_i2:_CTR1,_SIS1,_AtCTR1 | 1.085 | 1.602 |
|  | c89719_g1_i2:_EFE,_ACO4,_EAT1 | 1.378 | 0.814 |
|  | c90299_g4_i1:_CTR1,_SIS1,_AtCTR1 | 0.336 | 4.864 |
|  | c90616_g4_i1:_RAN-1,_RAN1,_ATRAN1 | 0.353 | 6.544 |
|  | c90729_g2_i1:_EIL2 | -1.047 | 2.295 |
|  | c91669_g2_i1:_LACS8 | 0.810 | 6.274 |
|  | c91804_g3_i1:_LACS8 | 0.721 | 2.760 |
|  | c91804_g3_i4:_LACS8 | 1.000 | 2.749 |
|  | c92062_g3_i2:_EFE,_ACO4,_EAT1 | 1.750 | -0.057 |
|  | c92603_g1_i1:_CTR1,_SIS1,_AtCTR1 | -0.611 | 3.704 |
|  | c92700_g4_i1:_CTR1,_SIS1,_AtCTR1 | 0.460 | 5.977 |
|  | c93034_g4_i1:_RAN-1,_RAN1,_ATRAN1 | 0.507 | 5.649 |
|  | c93557_g1_i3:_CTR1,_SIS1,_AtCTR1 | 0.749 | 2.402 |
|  | c93856_g1_i6:_CTR1,_SIS1,_AtCTR1 | 0.747 | 2.145 |
|  | c93875_g1_i2:_EFE,_ACO4,_EAT1 | 0.860 | 2.572 |
|  | c93875_g1_i3:_EFE,_ACO4,_EAT1 | 0.718 | 2.803 |
|  | c93875_g1_i4:_EFE,_ACO4,_EAT1 | 1.298 | 2.528 |
|  | c94636_g1_i1:_CTR1,_SIS1,_AtCTR1 | 0.795 | 2.870 |
|  | c94812_g1_i3:_ERF1,_ATERF1 | 0.704 | 2.875 |
|  | c94812_g1_i4:_ERF1,_ATERF1 | 0.628 | 3.259 |
|  | c95178_g4_i1:_CTR1,_SIS1,_AtCTR1 | 0.523 | 4.407 |
|  | c96777_g1_i1:_LACS8 | -1.691 | 0.546 |
|  | c96924_g3_i3:_CTR1,_SIS1,_AtCTR1 | 0.975 | 2.245 |
|  | c96924_g3_i5:_CTR1,_SIS1,_AtCTR1 | 0.863 | 3.730 |
|  | c96924_g3_i9:_CTR1,_SIS1,_AtCTR1 | 1.248 | 2.032 |
|  | c97864_g1_i1:_CTR1,_SIS1,_AtCTR1 | 0.627 | 3.798 |
|  | c98667_g1_i1:_CTR1,_SIS1,_AtCTR1 | -0.919 | 3.384 |
|  | c98667_g1_i2:_CTR1,_SIS1,_AtCTR1 | -1.033 | 3.516 |
|  | c98667_g1_i3:_CTR1,_SIS1,_AtCTR1 | -0.746 | 3.149 |
|  | c99075_g7_i5:_LACS8 | 1.238 | 1.527 |
