# Supplementary material for: Transcriptomic changes reveal gene networks responding to the overexpression of a blueberry DWARF AND DELAYED FLOWERING 1 gene in transgenic blueberry plants
Source: BMC Plant Biol. 2017 Jun 19;17:106. doi: 10.1186/s12870-017-1053-z (PMC5477172; doi:10.1186/s12870-017-1053-z)
Supplement: Supplementary file 3 — Table S3. Primers used for RT-PCR. FDR (false discovery rate) < 0.05. LogFC: log2(fold change) = Log2(Legacy-VcDDF1-OX/Legacy) (DOCX 76 kb) [file 12870_2017_1053_MOESM3_ESM.docx]

**Table S3.** Primers used for RT-PCR. LogFC: log_2_(fold change) =Log_2_(Legacy-VcDDF1-OX/Legacy)

| Primer name | Primer (5' to 3' end) | | | LogFC | | | |
| --- | --- | --- | --- | --- | --- | --- | --- |
| c91063_g2_i2:_ARR18,_RR18 FWD | | AAG GAA GAG GAG GAA GAA GAA | | | 1.35 | |  |
| c91063_g2_i2:_ARR18,_RR18 REV | | GGC ATG GTT AGG TTG GAA TAG | | | 1.35 | |  |
| c92839_g2_i5:_KAO2 FWD | | CCA ACA TGA TGA GGT TAG AGA A | | | 1.69 | |  |
| c92839_g2_i5:_KAO2 REV | | GGA GAC TGA GTG AGG TAG AA | | | 1.69 | |  |
| c93443_g1_i2:_CYP83B1,_SUR2,_RNT1,_RED1,_ATR4 FWD | | ACT CAT CCT ATC CTC TCC TTC | | | 1.80 | |  |
| c93443_g1_i2:_CYP83B1,_SUR2,_RNT1,_RED1,_ATR4 REV | | CTC TTC TCT ACT TTC CCG TAA TC | | | 1.80 | |  |
| c93875_g1_i4:_EFE,_ACO4,_EAT1 FWD | | GCC TCC ATT CCT TCT AAC TAC | | | -1.30 | |  |
| c93875_g1_i4:_EFE,_ACO4,_EAT1 REV | | CTT CCA ATC CTA AGC CCT TC | | | -1.30 | |  |
| c95166_g2_i6:_CYP88A3,_ATKAO1,_KAO1 FWD | | GAT CTC GCG GAG GTA ATA AAG | | | -1.68 | |  |
| c95166_g2_i6:_CYP88A3,_ATKAO1,_KAO1 REV | | GCC CTT TGA ATC CGT CTA AT | | | -1.68 | |  |
| c95687_g3_i1:_CYP83B1,_SUR2,_RNT1,_RED1,_ATR4 FWD | | CAC CAG CAA CAC TCC TAA TC | | | -1.17 | |  |
| c95687_g3_i1:_CYP83B1,_SUR2,_RNT1,_RED1,_ATR4 REV | | GTC TTT CGG TAG GAA ACC ATC | | | -1.17 | |  |
| C94438_g3_i2: Eukaryotic translation initiation factor 3 subunit H FWD | | GAGAGATTCAGATGCCCAGAAG | | |  | |  |
| C94438_g3_i2: Eukaryotic translation initiation factor 3 subunit H REV | | GGACAATGGATGGACCAGATT | | |  | |  |
|  | | |  | | |  |  |
|  | | |  | | |  |  |
