# Supplementary figures and images for: Transcriptomic changes reveal gene networks responding to the overexpression of a blueberry DWARF AND DELAYED FLOWERING 1 gene in transgenic blueberry plants
Source: BMC Plant Biol. 2017 Jun 19;17:106. doi: 10.1186/s12870-017-1053-z (PMC5477172; doi:10.1186/s12870-017-1053-z)

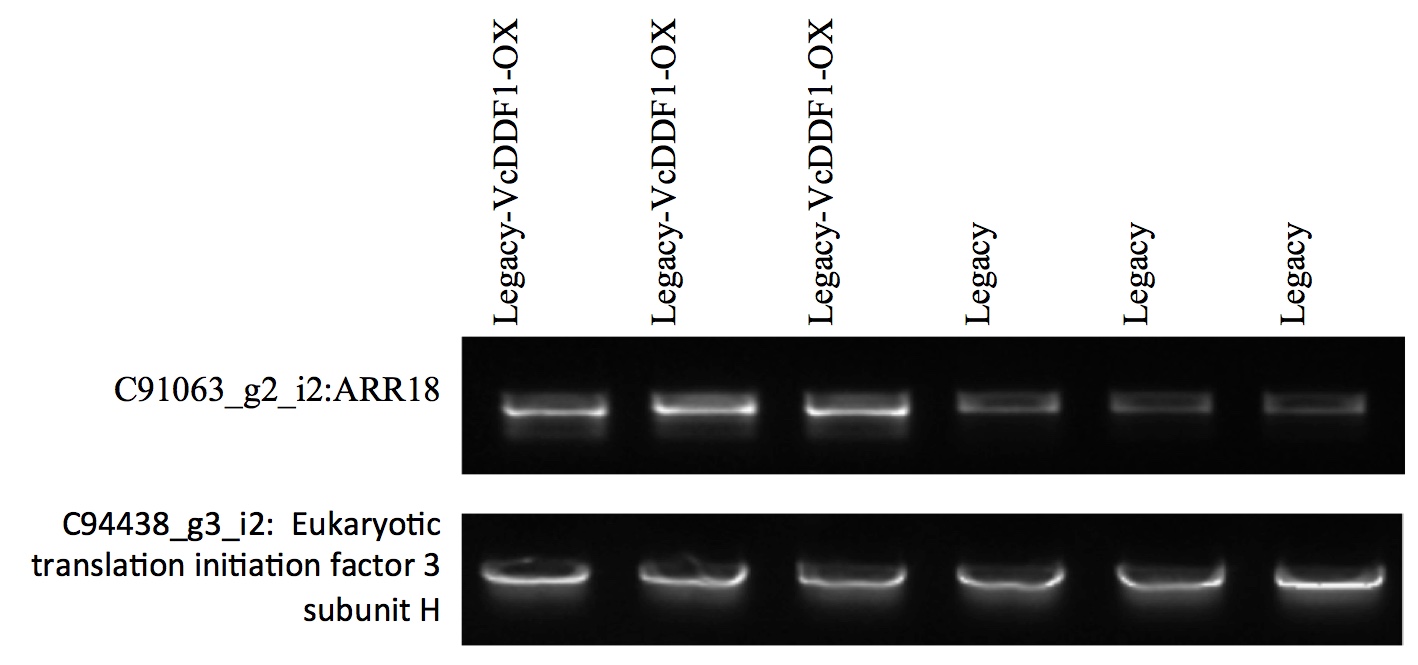

Supplement: Supplementary file 4 — Fig. S1. RT-PCR analysis of differentially expressed transcripts in leaf tissues of non-transgenic ‘Legacy’ and transgenic ‘Legacy-VcDDF1-OX’. Eukaryotic translation initiation factor 3 subunit H is the internal control (JPEG 119 kb) [file 12870_2017_1053_MOESM4_ESM.jpeg]
