# Supplementary material for: Transcriptomic changes reveal gene networks responding to the overexpression of a blueberry DWARF AND DELAYED FLOWERING 1 gene in transgenic blueberry plants
Source: BMC Plant Biol. 2017 Jun 19;17:106. doi: 10.1186/s12870-017-1053-z (PMC5477172; doi:10.1186/s12870-017-1053-z)
Supplement: Supplementary file 5 — Table S4. The pathway genes of major phytohormones [i.e., gibberellin (GA) [75], abscisic acid (ABA) [75], cytokinin [76], indole acetic acid (IAA) [77], ethylene [78], and DELLA protein genes in A. thaliana (DOCX 89 kb) [file 12870_2017_1053_MOESM5_ESM.docx]

**Table S4.** The pathway genes of major phytohormones [*i.e.,* gibberellin (GA) [68], abscisic acid (ABA) [69], cytokinin [70], indole acetic acid (IAA) [71], ethylene [72], and DELLA protein genes in *A. thaliana*.

|  | Gene identity | Gene function |
| --- | --- | --- |
| DELLA |  |  |
|  | AT1G14920.1 | Member of the DELLA proteins that restrain the cell proliferation and expansion that drives plant growth. |
|  | AT1G66350.1 | Negative regulator of GA responses |
|  | AT2G01570.1 | Member of the VHIID/DELLA regulatory family. |
|  | AT3G03450.1 | Encodes a DELLA protein |
|  | AT5G17490.1 | DELLA subfamily member involved in GA signal transduction |
| ABA |  |  |
|  | At5g67030 | *ZEP* |
|  | At1g52340 | *SDR1* |
|  | At3g14440 | *NCED3* |
|  | At1g78390 | *NCED5* |
|  | At1g16540 | *MCSU* |
| Cytokinin: ARR (A-type and B-type responsive regulator) |  |  |
|  | A-type |  |
|  | At1g59940 | ARR3 |
|  | At1g10470 | ARR4 (ATRR1 and IBC7) |
|  | At3g48100 | ARR5 (ATRR2 and IBC6) |
|  | At5g62920 | ARR6 |
|  | At1g19050 | ARR7 |
|  | At2g41310 | ARR8 (ATRR3) |
|  | At3g57040 | ARR9 (ATRR4) |
|  | At1g74890 | ARR15 |
|  | At2g40670 | ARR16 |
|  | At3g56380 | ARR17 |
|  | At3g04280 | ARR22 |
|  | B-type |  |
|  | At3g16857 | ARR1 |
|  | At4g16110 | ARR2 (ARP5) |
|  | At4g31920 | ARR10 (ARP4) |
|  | At1g67710 | ARR11 (ARP3) |
|  | At2g25180 | ARR12 |
|  | At2g27070 | ARR13 |
|  | At2g01760 | ARR14 |
|  | At5g58080 | ARR18 |
|  | At1g49190 | ARR19 |
|  | At3g62670 | ARR20 |
|  | At5g07210 | ARR21 |
|  | At5g62120 | ARR23 |
|  | Response regulator-like protein |  |
|  | At5g61380 | APRR1 (TOC1) |
|  | At4g18020 | APRR2 |
|  | At5g60100 | APRR3 |
|  | At5g49240 | APRR4 |
|  | At5g24470 | APRR5 |
|  | At1g68210 | APRR6 |
|  | At5g02810 | APRR7 |
|  | At4g00760 | APRR8 |
|  | At2g46790 | APRR9 (TL1) |
| GA |  |  |
|  | At5g25900 | KO1 |
|  | 1g15550 | GA3ox1 |
|  | A5g51810, | GA20ox2 |
|  | At1g02400 | GA2ox4 |
|  | At1g05160 | KAO1 |
|  | At1g30040 | GA2ox2 |
|  | At1g78440 | GA2ox1 |
|  | At1g80340 | GA3ox2 |
|  | At2g32440 | KAO2 |
|  | At2g34550 | GA2ox3 |
|  | At4g25420 | GA20ox1 |
|  | At5g07200 | GA20ox3 |
| Ethylene |  |  |
|  | AT1G66340 | ETR1 |
|  | AT3G23150 | ETR2 |
|  | AT2G40940 | ERS1 |
|  | AT1G04310 | ERS2 |
|  | AT3G04580 | EIN4 |
|  | AT1G54490 | EIN5 |
|  | AT3G20770 | EIN3 |
|  | AT5G03280 | EIN2 |
|  | AT5G03730 | CTR1 |
|  | AT5G20010 | RAN1 |
|  | AT1G25490 | EER1 |
|  | AT3G23240 | ERF1 |
|  | AT2G27050 | EIL1 |
|  | AT5G21120 | EIL2 |
|  | AT4G37580 | HLS1 |
|  | AT3G61510 | ACS1 |
|  | AT1G01480 | ACS2 |
|  | AT1G64400 | ACS3 |
|  | AT2G22810 | ACS4 |
|  | AT3G51770 | ACS5 |
|  | AT4G11280 | ACS6 |
|  | AT2G04350 | ACS8 |
|  | AT3G49700 | ACS9 |
|  | AT1G62960 | ACS10 |
|  | AT4G08040 | ACS11 |
|  | AT1G05010 | ACO |
| IAA |  |  |
|  | AT3G44310 | NIT1 |
|  | AT3G44300 | NIT2 |
|  | AT5G20960 | AtAO1 |
|  | AT3G02875 | ILR1 |
|  | AT1G51760 | IAR3 |
|  | AF301899 | RTY |
|  | AT4G31500 | SUR2 |
|  | AT2G38120 | AUX1 |
|  | AT1G73590 | PIN1 |
|  | AT5G57090 | EIR1 |

| \|  \|  \| \| --- \| --- \| \| EIN6 \| EIN6 \| \| ISR1 \| ISR1 \| \| ACS7 \|  \| \| ACS12 \|  \| \|  \|  \| |  |
| --- | --- | --- | --- | --- | --- | --- | --- | --- | --- | --- | --- | --- | --- |
|  |  |
|  |  |
|  |  |
|  |  |
|  |  |
|  |  |
|  |  |
|  |  |
